# Towards equity? The trajectory of women’s participation in the summer paralympic games (1988–2024)

**DOI:** 10.3389/fspor.2025.1593956

**Published:** 2025-06-30

**Authors:** Luiz Gustavo Santos, Beatriz Lucena Ramos, Vânia Tie Koga Ferreira, Ruth Eugênia Cidade, Ciro Winckler

**Affiliations:** ^1^ Sports Development Department, Brazilian Paralympic Committee, São Paulo, Brazil; ^2^ Parasport Study Center, São Paulo Federal University, Santos, Brazil; ^3^ Parasport Secretary, Brazil Sport Ministry, Brasília, Brazil; ^4^ Physical Education Department, Paraná Federal University, Curitiba, Paraná, Brazil

**Keywords:** women, paralympic, inclusion, disability, sport disability, Parasport

## Abstract

**Introduction:**

Women's participation in the Paralympic Games has increased over recent decades, yet significant challenges persist. This study examines the trajectory of female athletes in the Summer Paralympic Games from 1988 to 2024, focusing on numerical growth and regional distribution.

**Methods:**

Employing a descriptive and comparative approach, data from the International Paralympic Committee were analyzed to assess participation by gender and continent across different editions of the Games.

**Results:**

The results show a substantial increase in female representation, rising from 22.06% in 1988 to 44.48% in 2024. However, this progress has not been uniform.

**Discussion:**

While Asia has experienced significant growth, Europe and the Americas saw a proportional decline in female participation. Africa, despite some progress, continues to have limited numbers. Additionally, the number of National Paralympic Committees without female athletes has increased, highlighting structural and sociocultural inequalities. The findings suggest that, although initiatives such as adopting the Brighton Declaration and expanding women's events have driven progress, barriers remain. The study concludes that region-specific policies are essential for advancing gender equity in Parasport, ensuring greater access and opportunities for women with disabilities in high-performance competition.

## Introduction

The origin of the Paralympic movement dates to World War II. In 1944, Ludwig Guttmann implemented a rehabilitation center for people with spinal cord injuries at Stoke Mandeville Hospital in the United Kingdom. At this center, sports activities were established as part of the physical and psychological rehabilitation program, also aiming at the social reintegration of war veterans (1). As part of this process, on 29 July 1948, the same day as the opening of the 2012 London Olympic Games, the first Stoke Mandeville Games were organized, with the participation of 16 military athletes, six of whom were women, competing in wheelchair archery (2).

Over the decades, the Paralympic Games have grown in size and scope, with an increasing number of athletes and countries participating. Until 1972, the Games were exclusive to individuals with spinal cord injuries (3). After the 1972 Games, athletes with visual, intellectual, and other impairments, such as upper or lower limb deficiencies, coordination impairments, short stature, and impaired muscle power, were included in subsequent Games (3). Concurrently, female participation also increased considerably. In the first Summer Paralympic Games (SPG), held in 1960, until 1980, few events were destined for women, resulting in a limited number of female competitors. Since the 1980s, more events for women were introduced, resulting in a steady rise in female participation in each SPG (3).The underrepresentation of women in the Paralympic Games reflects a broader historical trend also present in the Olympic Games, where female participation was limited until intentional gender equity policies were enacted in the late 20th and early 21st centuries (4).

Recognizing these disparities, various international initiatives emerged to promote gender equity in sport. Among them, the Brighton Declaration was established in 1994 by the International Working Group on Women and Sport, representing a major milestone. The Declaration set fundamental principles aimed at increasing women's participation, leadership, and recognition across all levels of sport. It was updated in 2014, becoming the Brighton Plus Helsinki 2014 Declaration, expanding its scope to include physical activity as an essential dimension of organized sports. The revised Declaration emphasized the need for a sports culture that enables and values women's full participation in all aspects of sport and physical activity (5).

In line with these global efforts, the International Paralympic Committee (IPC) formally adopted the Brighton Declaration in 1997 (6). The first event following the adoption of the Declaration was the Sydney 2000 Paralympic Games, where women comprised 25% of the participants, representing 65% of the participating countries. Of the 20 sports contested, 15 included female participation (7).

Aligned with this initiative, the IPC Women in Sport Committee was established in 2003 with advisory and consultative responsibilities to implement actions to increase female athletes, coaches, officials, and leadership positions (8). These initiatives led to the implementation of more effective measures to increase female participation, such as improving event qualification criteria, increasing the number of women's events, and adopting different models for analyzing international rankings (9). In the 2012 Games, sports such as rowing and equestrian had already achieved equity values (50% and 71%, respectively). However, sports such as wheelchair rugby (2.2%) and sailing (18.8%) still exhibited significant disparities. Both disciplines are officially classified as mixed-gender by the IPC; however, they demonstrate a noticeable predominance of male participation in practice (9). At the Tokyo 2020 Games, women comprised approximately 40% of Paralympic athletes, while at the at the PyeongChang 2018 Winter Games, the disparity was more evident, with only 23% female participation (10).

Gender equity has not yet been fully achieved. Despite the increase in women's events and the implementation of participation quotas by the IPC, significant disparities highlight specific structural challenges for athletes worldwide (11). The study by Oggero et al. (11), which analyzed the Gross National Income per capita of countries participating in the Paralympic Games from 1960 to 2016, found no differences in female participation among different economic groups, indicating that the primary barrier lies in cultural factors. Historically, women with disabilities have faced greater challenges than men in accessing sports opportunities, often due to persistent social and cultural barriers such as gender stereotypes, discrimination based on disability and gender, and lack of family and community support for women's participation in sports, and limited access to leadership roles and decision-making positions within sports organizations (12).

According to estimates from the United Nations, the world population has reached 8 billion people, distributed as follows: Asia (59%), Africa (17%), the Americas (13%), Europe (9%), and Oceania (0.5%). The gender distribution remains relatively balanced: Asia (51.0% male/49.0% female), Africa (50.2%/49.8%), the Americas (49.5%/50.5%), Europe (48.5%/51.5%), and Oceania (50.5%/49.5%) (13). Data from the WHO indicate that 16% of the global population has at least one type of disability, with 14.2% among men and 18% among women (14). The estimated percentage of people with disabilities varies across region: Asia (15%), Europe (16%), Oceania (17%), the Americas (12%), and Africa (10%) (14). The number of countries per continent, as recognised by the United Nations, is 54 in Africa, 35 in the Americas, 50 in Asia, 57 in Europe, and 14 in Oceania (13). These demographic distributions underscore the importance of ensuring gender equity in international sporting movements like the Paralympic Games. Given the relatively balanced global gender distribution and the proportion of women with disabilities worldwide, promoting equitable opportunities for female athletes is not only a matter of fairness but also of representativeness and inclusivity. In this context, gender equity has increasingly become a central theme within the Paralympic Movement (15), leading to sustained efforts to promote equal opportunities for all athletes, regardless of gender (12).

While global initiatives like the Brighton Declaration and IPC policies have advanced gender equity in Parasport, the effects of these measures can vary across regions due to structural, cultural, and economic differences. Differences in national investment, cultural norms, and access to the sports infrastructure impact the representation of women with disabilities in high-performance Parasports across continents. Therefore, understanding regional patterns is essential for identifying disparities and guiding the development of context-specific strategies beyond global declarations.

The aim of this study was to analyze the evolution of women's participation in the Summer Paralympic Games from 1988 to 2024, with particular attention to the regional distribution of athletes. This includes examining differences in female participation by continent, identifying trends in the number of National Paralympic Committees (NPCs) that did not include female athletes, and assessing each region's representation over time.

## Materials and methods

This is a descriptive and comparative study with a longitudinal design. The data used in this study were obtained from the IPC website (https://www.paralympic.org/) and the official IPC results books covering the years 1988 to 2024, corresponding to the modern era of SPG (2). The number of participating athletes of both genders was extracted globally. All data were tabulated and analyzed using Microsoft Excel®. Subsequently, the data were grouped by gender, the continent to which each National Paralympic Committee (NPC) belongs (Africa, Americas, Asia, Europe, Oceania), and the year of participation. Descriptive data analysis was conducted using absolute values regarding the number of participants in each SPG. The data were presented in graphical format.

### Definition of variables

#### Participants

Athletes registered in the event results book.

#### Number of NPCs without women

NPCs per continent that did not have registered female participants in the analyzed Games.

#### % of participants distributed by gender by games

The number of participants by gender was relativized by the total number of participants in each SPG using specific equations:

• Equation 1: % of male participants per Games -(%PMxSPG):
  (%PMxSPG=(nmaleparticipants*100))/(nTotalGameparticipation)
• Equation 2: % of female participants per Games -(%PWxSPG):
  (%PWxSPG=(nfemaleparticipants*100))/(nTotalGameparticipation)

#### % of women distributed by continent by games

The number of female participants per continent was relativized by the total number of participants in each SPG:

• Equation 3:
  $$\begin{array}{r} (\text{\%}\mathrm{PWxCSPG}=\\ \;\left(n\;\mathrm{female}\;\mathrm{participants}\;(ex:\mathrm{America})*100\right))/(n\;\mathrm{Total}\;\mathrm{Game}\;\mathrm{participation}) \end{array}$$

## Results

**Figure 1:**
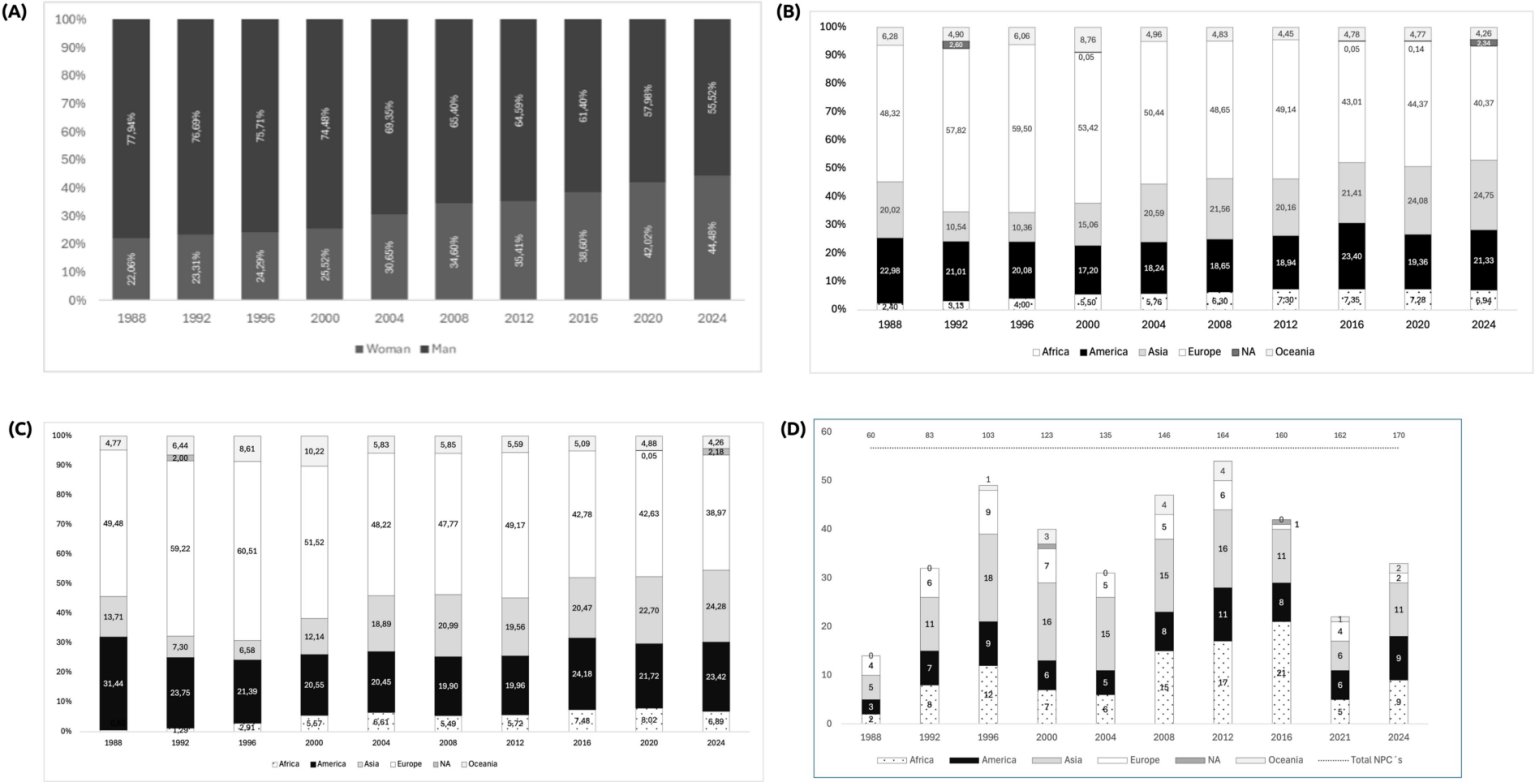
**(A)** % participants distributed by gender and edition of summer paralympic games - SPG **(B)** % participants (Men and women) distributed by continent and edition of SPG **(C)** % of women distributed by continent and edition of SPG and **(D)** number of NPCs without women distributed by continent and edition of SPG.

The data presented in the four graphs shows the evolution of women's participation over the analyzed period, as well as the characteristics of regional women's participation in the SPG (Figure 1).

## Discussion

The evolution of athlete participation in the SPG has grown from 400 athletes in the first Games in Rome in 1960 to 4,433 athletes in the Paris 2024 Games. The data analysis reveals an increase in female participation in the SPG over the modern era (G1). In 1988, women's representation was 22.06%, whereas in 2024, this figure reached 44.48%, representing an increase of 22.42% over 36 years. From 2008 onwards, this growth intensified, with female participation rising from 34.60% to 44.48% in 2024, equivalent to an increase of 9.88%, driven by measures adopted since the early 2000s (6).

Although the adoption of the Brighton Declaration by the IPC represented a key commitment to gender equity, our study did not directly evaluate the specific effects of this initiative on women's participation rates. Rather, our findings document general participation trends over time, which may have been influenced by a range of factors, among them the principles promoted by the Brighton Declaration.

Another key factor in this progress was the introduction of sports with balanced participation between men and women, class adjustments, and medal events. Regarding sports balance, women's competitions were introduced in previously male-only events (Cycling—1992, Powerlifting—2000, Sitting Volleyball and Judo—2004) (9), as well as the separation of mixed events into male and female categories (Boccia—2024), the exclusion of predominantly male events (Sailing—2020), and male-exclusive events (Standing Volleyball—2004 and CP Football—2020). However, mixed-gender sports with a male predominance were introduced (Wheelchair Rugby—2000) (16), as well as a male-exclusive sport (Blind Football—2004) (17). However, the sports disciplines introduced since 2008 are characterized by a balance between male and female events (Rowing, Canoe, Badminton, Taekwondo, and Triathlon) (10, 11). In pursuit of equity, other individual sports disciplines (e.g., athletics, swimming, and cycling) have been adjusting the number of medal events to achieve gender balance (10). Notably, equestrian stands out as a discipline where women not only participate equally but actually outnumber men. In both the Tokyo 2020 and Paris 2024 Paralympic Games, the majority of equestrian competitors were women, reinforcing the idea that the structural and cultural characteristics of specific sports can create favorable conditions for female representation (18).

The analysis of the distribution of participants in the Summer Paralympic Games between 1988 and 2024 highlighted the overall increase in the geographical diversity of competitors. The percentage of athletes from Asia among the total participants steadily increased after hosting the 1988 Games, rising from 10.54% in 1992 to 24.75% in 2024. This growth may be related to a combination of factors, including efforts to expand regional training infrastructure, the introduction of new sports events for women, and broader inclusion strategies, possibly implemented by National Paralympic Committees. However, our dataset does not allow us to isolate or confirm the effect of any specific policy or investment. On the other hand, the percentage of athletes from the Americas showed a slight decline, varying from 22.98% in 1988 to 21.33% in 2024, with the highest participation recorded during the Games in Brazil. Although Europe had the highest percentage of representation in earlier editions (59.50% in 1996), its share gradually decreased over time, reaching 40.37% in 2024. Africa demonstrated gradual growth in its percentage of participants, increasing from 2.40% in 1988 to 6.94% in 2024, reflecting a broader inclusion trend, although participation levels remain modest. Oceania's percentage decreased compared to its initial values; however, since the Sydney 2000 Games, it has remained stable, maintaining a participation rate of 4.26% in 2024.

From a macro perspective, where actions aimed at enhancing diversity in the SPG are considered, there is a broader distribution alongside a reduction in the percentage of participation from Europe. However, when accounting for each region's population and number of countries, these patterns indicate that inclusion policies and investments in Paralympic sports have had a impact, particularly in Asia. Simultaneously, they suggest a proportional decrease in participation from Europe and the Americas compared to other regions. Furthermore, when analysing the distribution in relation to the number of countries, it becomes evident that Africa and Asia remain considerably below their potential in terms of participation. This is particularly relevant given that each country has a maximum number of athletes who can qualify, alongside the overall population of these continents (19).

In G3, female participation showed fluctuations throughout the analysed period. Asia distinguished itself with notable growth, increasing from 13.71% in 1988 to 24.28% in 2024, reflecting substantial investment and development in Paralympic sports. According to Brittain (9), Asian NPCs have experienced increased female athletes over the years, indicating a more pronounced effort toward female inclusion linked to the movement to host major Games. Despite barriers in certain countries, there is a growing recognition of the importance of female participation in sport as a tool for social empowerment and inclusion (10). Conversely, Europe, which initially had the highest female participation (49.48% in 1988), experienced a decrease of 10.52% by 2024. These data point to the effects of the decentralisation of Parasport in relation to Europe, a trend previously identified by Brittain (9) in his analysis of the 2012 Paralympic Games. This process has intensified, revealing a similar pattern in female participation, showcasing greater representation from other regions without an absolute decline in European participants.

Another pertinent aspect of this analysis is the suspension of Russia, which may have contributed to an underestimation of Europe's participation in 2024 (20). Africa recorded the lowest female participation, however, it gradually increased from 0.60% in 1988 to 6.89% in 2024, signifying progress in supporting women's sports. These figures may be partially associated with the efforts of development programs led by the IPC and the Agitos Foundation, which have aimed to increase access to Parasport in underrepresented regions through initiatives such as equipment donation, coach education, athlete training camps, and capacity-building for NPCs (9). However, as our analysis is based on descriptive participation data, we cannot determine how these programs directly influenced the observed trends. Furthermore, existing literature points to various persistent challenges — including economic constraints, sociocultural norms, and limited infrastructure — that may hinder progress in several African countries (10). Lastly, Oceania maintained a stable participation rate, declining slightly from 4.77% in 1988 to 4.26% in 2024. This region's statistics may be attributed to its smaller population and, consequently, a limited athletic base. While Australia and New Zealand boast strong sports inclusion policies, other smaller Pacific nations may not have the same investment and institutional support (9).

In summary, while Asia and Africa experienced increases, Europe and the Americas saw a decline in the proportion of female participants during this period. The analysis of NPCs without female representatives in the SPG illustrates progress and ongoing challenges in promoting women's inclusion across the editions. It also reflects the evolution of Paralympic sport across each continent based on male participation, as noted in G4. The lack of inclusive sports policies and insufficient financial and institutional incentives greatly contribute to the disparity in female representation across continents (9, 11). Furthermore, the unequal allocation of resources for adaptive sports directly affects the development and retention of female athletes in international competitions (21). In 1988, only 3.3% of NPCs did not send female athletes; by 2024, this proportion had increased to 5.3%. In the Americas, the number of NPCs without female representatives rose from 3 in 1988 to 9 in 2024. Similarly, the number of NPCs without female athletes in Africa increased from 3 in 1988 to 9 in 2024. Asia also witnessed a rise in NPCs without female participants, from 5 in 1988 to 11 in 2024.

Sociocultural, structural and systemic barriers continue to restrict access to Parasports for women with disabilities. Gender stereotypes and cultural norms often limit sporting opportunities, especially for women with disabilities, who face intersecting forms of discrimination and are frequently excluded from economic incentive programmes designed to foster sports participation (10, 22). These limitations are compounded by the lack of accessible infrastructure—such as adapted sports facilities and reliable transportation—and by the scarcity of coaches with training to support athletes with diverse impairments (23). Moreover, additional factors such as geographical isolation, restrictive religious or cultural norms, and institutional neglect hinder access and continuity in sports trajectories. Structural issues internal to the Paralympic Movement, particularly within the classification system, still result in the exclusion of certain impairment types from official recognition or competitive opportunities, thereby perpetuating inequality in representation and participation (24, 25). Systemic underdevelopment of grassroots and school-based sports programmes—especially in low- and middle-income countries—has also been identified as a critical limitation for identifying and developing female athletes (10). These interrelated barriers reflect persistent equity gaps, revealing the need for inclusive and context-sensitive policies that recognize impairment diversity, regional and cultural plurality, and the gendered dimensions of access. Addressing these issues is essential to realizing the transformative goals promoted by the Paralympic Movement.

In contrast to the previously presented scenario, Europe has demonstrated progress, with the number of NPCs without female representatives decreasing from four in 1988 to two in 2024. This reflects advancements in the inclusion of women in the SPG. The higher levels of female participation observed in Europe may be partially associated with broader social and institutional developments in the region. Although our study did not directly analyze public policies or investment patterns, existing literature has pointed to the relevance of national policy frameworks, stable funding, and grassroots initiatives in promoting women's access to sport (26). Initiatives, such as the introduction of quotas for female athletes in competitions, the promotion of female coaches and sports managers within the Paralympic movement, and awareness campaigns regarding gender equity, have been shown to be effective strategies for boosting female participation across Europe (27, 28).

These findings must also be understood in the context of broader international commitments to gender equity in sport, such as those outlined in the Brighton Declaration on Women and Sport (5), which was reaffirmed globally through the Brighton + Helsinki 2014 Update. While the Declaration calls for equitable opportunities, resources, and access for women in sports—regardless of region or disability—our analysis reveals persistent disparities in participation, particularly outside Europe. The ongoing underrepresentation of women in certain regions indicates that implementing the Declaration's principles is still inconsistent, especially in the Paralympic context. These results, therefore, highlight the need for greater alignment between global advocacy instruments and national policy and development strategies to ensure inclusive and effective implementation.

This study has several limitations that must be acknowledged. First, it relies solely on publicly available secondary data from the IPC, which restricts the depth of contextual information regarding national policies, institutional structures, or athlete development pathways. Second, our descriptive analysis cannot establish causal relationships between regional patterns and specific social or policy factors. Third, we did not disaggregate participation data by type of impairment, sport, or age group, which may obscure intersectional disparities. Considering the current gender disparities in team sports and the predominance of male participation in mixed-gender disciplines, it is worth highlighting a structural limitation within the Paralympic program: the absence of collective sports exclusively for women beyond sitting volleyball and goalball. Considering that eight of the IPC's Minimum Impairment Criteria (MIC) are associated with physical disabilities, it is reasonable to suggest that any potential addition of a female-only team sport should be aligned with these impairment classes. Lastly, while we reference international policy frameworks such as the Brighton Declaration, our study does not empirically assess their implementation or effectiveness across regions. These limitations indicate that further qualitative and mixed-methods research is necessary to explore the underlying mechanisms driving the observed trends.

## Conclusion

The results indicate increased female participation throughout the SPG, with a better distribution across continents. That said, regional disparities remain. Moreover, collaboration between governments, Paralympic committees, and civil society organisations must be strengthened to promote access to sports and solidify the presence of women in high-performance competitions. This scenario highlights the need for further adjustments and the development of policies tailored to each country's regional and cultural characteristics rather than relying solely on centralized actions by the International Paralympic Committee.

## Data Availability

The raw data supporting the conclusions of this article will be made available by the authors, without undue reservation.
